# Social Cognitive Dysfunction in Elderly Patients After Anesthesia and Surgery

**DOI:** 10.3389/fpsyg.2020.541161

**Published:** 2020-09-24

**Authors:** Delin Zhang, Jun Ying, Xiaochi Ma, Zaifeng Gao, Hanjian Chen, Shengmei Zhu, Liping Shi, Xiqian Lu

**Affiliations:** ^1^Department of Anesthesiology, First Affiliated Hospital, School of Medicine of Zhejiang University, Hangzhou, China; ^2^Department of Gynaecology, First Affiliated Hospital, School of Medicine of Zhejiang University, Hangzhou, China; ^3^Department of Psychology and Behavioral Sciences, Zhejiang University, Hangzhou, China; ^4^Institute of Psychology, Chinese Academy of Sciences, Beijing, China

**Keywords:** social cognition, biological motion, holistic processing, anesthesia and surgery, delayed neurocognitive recovery

## Abstract

Extensive studies have revealed that cognitive processing was impaired after anesthesia and surgery, particularly for the elderly patients. However, most of the existing studies focused on the general cognitive deficits (e.g., delayed neuro-cognitive recovery and POCD). Although diagnosis of social abilities has been used in various clinical fields, few studies have investigated the potential deficit on social cognition after anesthesia and surgery. The current study examined whether there was any social cognitive dysfunction after anesthesia and surgery. We achieved this by taking biological motion (BM) as the stimuli of interest, the perception of which has been taken as the hallmark of social cognition. The elderly patients (aged ≥ 60 years) were required to judge whether an upright BM stimulus appeared among the dynamic noises to test their social cognition, as well as do a Mini-Mental State Examination to test their general cognition. The two tests were performed at both 1-day before and 7-day after the surgery. Results showed that 31.25% of patients exhibited BM perception deficit after anesthesia and surgery relative to before anesthesia and surgery, implying that social cognitive dysfunction existed. Meanwhile, social cognitive dysfunction was independent from delayed neurocognitive recovery.

## Introduction

Mental states of patients can be influenced after anesthesia and surgery, particularly for the elderly patients (Moller et al., 1998; Evered et al., 2018). To date researchers have mainly focused on cognitive dysfunction, including memory loss, inattention, and difficulties in processing information (Hovens et al., 2012). These symptoms are summarized as delayed neurocognitive recovery (within 30 days after anesthesia and surgery) or post-operative cognitive dysfunction (POCD) (1–12 months later after anesthesia and surgery) (Moller et al., 1998; Krenk et al., 2010; Evered et al., 2018). Despite there have been abundant evidence revealing the deficit of general cognitive ability after anesthesia and surgery, little is known about the influence of anesthesia and surgery on social cognition.

Human beings are extensively social in nature. Social cognition, the processes to perceive, manipulate and interpret social information (Henry et al., 2015), is vital to the quality of daily life (Hasson-Ohayon et al., 2017; Yogarajah and Mula, 2019). Ample studies have demonstrated that social cognition has independent neural substrates and functions from general cognitive ability (see Van Overwalle, 2009 for a review). However, currently most clinical studies have focused on the deficit of general cognitive ability (Hovens et al., 2012). Although a significant amount of studies implyed that patients with neurodegenerative disease (e.g., Alzheimer and Parkinson disease) have deficit in social cognition (for a review see, Christidi et al., 2018), to the best of our knowledge, there is no empirical evidence showing that the social ability is impaired after anesthesia and surgery. The present study attempted to close this gap, by addressing the following two questions: first, whether social ability could be impaired for elderly patients after anesthesia and surgery [i.e., social cognitive dysfunction (SCD)]; second, if SCD occurred, whether this social cognition deficit was independent of general cognition deficit (delayed neurocognitive recovery), which is measured *via* the Mini-Mental State Examination (MMSE, Folstein et al., 1975).

To explore the two questions, one key issue was to select a proper measurement that reflects one’s ability of social cognition. In the present study, we determined to use a perception task employing biological motion (BM) stimuli, which are movements of animate entities (e.g., walking, jumping, and waving by humans) (Johansson, 1973; Troje, 2013). The processing capability of BM has been considered as a hallmark of social cognition (Pavlova, 2012; Gao et al., 2016), and studies on BM have been among the most important and fruitful fields in social cognition in the last decade (e.g., Puce and Perrett, 2003; Blake and Shiffrar, 2007; Pavlova, 2012; Troje, 2013; Steel et al., 2015). The superior temporal sulcus and ventral premotor cortex, which have been implicated in various aspects of human social cognition (Puce and Perrett, 2003; Gallese et al., 2004; Blakemore, 2008, 2012; Burnett et al., 2011) and were the core neural substrates for social cognition, play a key role in BM processing (Puce and Perrett, 2003; Grossman et al., 2005; Blake and Shiffrar, 2007; Gilaie-Dotan et al., 2013; Troje and Aust, 2013; Gao et al., 2014; Lu et al., 2016).

Befitting the importance of perceiving human BM information, we were all experts at processing BM. Even infants during their first few days of life (e.g., 2 days) already showed preferential attention toward upright BM (Bardi et al., 2011, 2013; Simion et al., 2011). This incredible ability of kinetic processing could be conspicuously demonstrated by point-light displays (PLDs) that depict human activity through a simple set of light points (e.g., 12 points). These lights were placed at distinct joints of a moving body (Johansson, 1973). Although highly impoverished (e.g., texture, hair, and form cues are absent), these PLDs were rapidly recognized as coherent and meaningful movements once in motion. Moreover, ample pieces of core social information, such as gender, emotion, intention and walking direction, could be extracted from PLDs (Rizzolatti et al., 2001; Puce and Perrett, 2003; Blakemore, 2008, 2012). Conversely, individual with deficits in social cognition also has impairment on BM perception (Blake, 2003). Therefore, BM processing has been considered as one of the most fundamental aspects of social cognitive processes (Pavlova, 2012; Troje, 2013; Henry et al., 2015). Consequently, we predicted that if the BM perception performance dropped after anesthesia and surgery in elderly patients, their social cognition would also impair.

In the present study, we tested one key characteristic of BM perception: our vision system processes BM in a global manner, which was typically reflected in inversion effect (Bertenthal and Pinto, 1994). Inversion effect was the significant impairment in perception of inverted BM relative to upright BM (Dittrich, 1993; Pavlova and Sokolov, 2000, 2003; Shipley, 2003; Troje, 2003). This effect could be manifested by embedding a PLD-based BM in a set of randomly moving dots. Although the PLD and random dots were constructed using the same elements, observers could quickly discriminate upright BM from noises, but hardly discriminate inverted BM from noises (Bertenthal and Pinto, 1994; Shiffrar et al., 1997; Pinto and Shiffrar, 1999). We investigated whether the global processing of BM (indexed by the inversion effect of BM) was impaired after anesthesia and surgery by comparing inversion effect between pre-operation and post-operation.

The current study chose elderly patients (aged ≥ 60 years) as our target group. Elderly patients have a larger incidence in delayed neurocognitive recovery or POCD than other age groups (Evered et al., 2018). We consider the elderly patients may be also more prone to the deficit of social cognition. Moreover, since the deficit of social cognition will significantly impact the life of the elders, it has practical value to figure out whether social cognition changed in elderly patients after anesthesia and surgery.

## Materials and Methods

### Participants

A total of 46 patients with cardiac surgery were enrolled in the present study, who provided signed informed consent. The age of these patients ranged within 60–77 years old, and ASA status of these patients was less than or equal to III. Furthermore, each patient had an education of primary school level or above. Patients were excluded from the study in case of an incomplete procedure (nine patients) or a low MMSE pre-operative score (<20, five patients). Four of 46 patients died before surgery; 6 of 46 patients chose to abandon the test after surgery; 4 of 46 patients discharged within 6 days after surgery, before the time point to conduct the test after surgery. A total of 32 patients (18 males and 14 females) completed the procedure, and the average age of these patients was 66.8 ± 4.8 years old. All participants had normal color vision and normal or corrected-to-normal visual acuity. Patients were further divided into two group for subsequent analysis according to a specific criterion (see section “Diagnostic Criteria of Delayed Neurocognitive Recovery and SCD”): 10 (four males) patients in SCD group and 22 (14 males) patients in non-SCD group. The study was approved by the Research Ethics Board of Zhejiang University.

### Surgery and Anesthesia

All patients underwent elective cardiac surgery on cardiopulmonary bypass (CPB), except for one patient who skipped CPB. Standard monitoring was used, including electrocardiogram, pulse oximetry, capnography, invasive arterial blood pressure, central venous pressure monitoring, temperature, and depth of anesthesia. Anesthesia was induced with 0.04 mg/kg of midazolam, 0.3 mg/kg of etomidate and 1 μg/kg of sufentanil, and muscle relaxation was achieved with 0.2 mg/kg of *Cis*-atracurium. Anesthesia was maintained using 40–80 μg/kg⋅min of propofol and 0.15 mg/kg⋅h of atracurium, and the patient was intermittently administered with 0.5 μg/kg of sufentanil and 0.04 mg/kg of midazolam through the vein to maintain the appropriate anesthetic depth. After the induction of anesthesia, a urinary catheter, rectal temperature probes and nasopharyngeal temperature probes were placed.

After the administration of an initial bolus of heparin of 300–400 IU/kg to maintain the activated clotting time above 480 s, CPB was initiated and maintained according to a strict protocol with standardized cannulation sites, pump flow, blood gas management, and mean arterial pressure (MAP) and temperature targets. Arterial blood gasses were measured at 15–30 min on CPB, in order to maintain the partial pressure of arterial carbon dioxide at 35–40 mmHg using alpha-stat correction and partial pressure of arterial oxygen at 150–250 mmHg. Cold cardioplegia was administered for myocardial protection. The blood-to-crystalloid ratio was 4:1. Perfusion was maintained at pump flows of 2.2–2.4 L/min/m^2^ to maintain a MAP of 50–80 mmHg. The pumps for all patients were SIII (Stockert, Munich, Germany) roller pumps. The oxygenators were obtained from Sorin Monolyth (Mirandola, Italy). Red blood cells were transfused to maintain a hematocrit of 20–25% on CPB. Weaning from CPB was attempted once systemic temperature (central) was 36°C, using a previously described protocol. Systolic blood pressure was maintained at 100–130 mmHg. Arterial blood gasses were measured at 30-min intervals to maintain the partial pressure of arterial carbon dioxide at 35–40 mmHg, and the partial pressure of arterial oxygen at 150 mmHg. Red blood cells were transfused to maintain a hematocrit of 25–30% throughout the case. After weaning from CPB, protamine was applied, accordingly.

### Psychological Tests

The test battery included two parts. The first part used MMSE (Folstein et al., 1975) as the means of diagnosing delayed neurocognitive recovery (Shi et al., 2015; Kulason et al., 2017). The second part was a BM perception task adopted from Bertenthal and Pinto (1994). The test battery was measured 1 day before the surgery (pre-operative condition) and 7 days after the surgery (post-operative condition).

**FIGURE 1:**
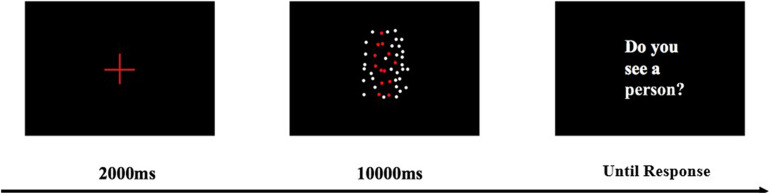
BM perception task with a scrambled mask. The red human figure is presented in white in the real experiment. Dynamic BM demos can be found in Supplementary Material.

After a 10-min paper-printed MMSE, patients completed a BM perception task that lasted for approximately 30 min. The BM task was run on a Lenovo Yoga 900 laptop. The screen size of this laptop was 13.3 inches, with a resolution of 3200 × 1800 and a refresh rate of 60 Hz. The patients sat 60 cm away from the screen of the laptop. For the BM perception task, participants were required to detect the walking human motion (8.08° × 14° in visual angle) masked by dynamic scrambled dots (Figure 1). All dots had the same size (0.28° × 0.28°) and color (white, RGB, [255, 255, 255]). These scrambled dots had the same trajectories as the BM joints, but the starting frame and location was randomized. The walking motion contained 43 frames and was played in a continuous loop at a rate of 60 frames per second. In each trial, the starting frame was fixed. The side-view walking BM consisted of 13 dots and was masked by 35 scrambled dots. When the walking BM was not presented, the BM was replaced by 13 scrambled BM dots. The stimulus was presented at the center of the screen, within a region of 11.2° × 20.8° of the visual angle. The background color was black (RGB, [0, 0, 0]). Trials were evenly split between present condition and absent condition, as well as between right orientation and left orientation. Before each trial began, a red (RGB, [255, 0, 0]) cross fixation (3.28° × 3.28°) was presented at the center of screen for 2 s. After the fixation disappeared, the stimulus was presented for up to 10 s until the patients orally reported whether there was a human walking. If a participant did not make a response within 10 s, an instruction “do you see a walking person” (in Chinese) was presented at the center of screen until an oral response was made. The inter-trial blank interval was 2 s. No feedback was displayed during the formal experiment. After every 20 trials, the patient should have a rest for at least 1 min. At the end of the break, the patient reviewed the stimuli and task requirements to avoid forgetting. Before the formal experiment, patients learned the side-view walking motion, and had five practice trials with no feedback, in order to familiarize themselves with the procedure.

The BM perception task was split into two blocks, according to the orientation of the BM. In the first block the BM was upright. The latter block was the inverted condition that every dot was inverted. Each block had 40 random trials, resulting in 80 trials in total. The sequence of these blocks was fixed, since our pilot study found that elderly patients had difficulties in recognizing the inverted pattern of BM when the inverted stimuli were taken as the first block.

### Demographics and Medical History

Both pre-operative factors and hospital associated factors, including age, education level, hypertension, diabetes, history of smoking, history of alcohol consumption, surgery duration, CPB duration, midazolam, sufentanil, time to ICU discharge and time to hospital discharge, were recorded.

### Diagnostic Criteria of Delayed Neurocognitive Recovery and SCD

We used the 1SD (one standard deviation) criterion for delayed neurocognitive recovery (Evered et al., 2011). According to the 1SD criterion, the MMSE difference between the pre-operative condition and post-operative condition was first calculated (score_*pre–operative*_ – score_*post–operative*_). If a patient’s MMSE decrease was higher than one standard deviation of pre-operative MMSE scores of all patients, that patient would be diagnosed as delayed neurocognitive recovery.

Since the present study was the first to empirically examine SCD, the 1SD criterion was adopted to have a direct comparison with delayed neurocognitive recovery. The performance of the BM perception task was calculated in terms of the signal detection theory, focusing on *d*′. As a robust sensitivity index for perception, *d*′ was calculated in both upright condition and inverted condition. Inversion effect (*d*′_*upright*_ – *d*′_*inverted*_) could represent the global processing ability of BM. Particularly, the potential reduction of inversion effect between pre-operative and post-operative conditions was first calculated (Inversion Effect_*pre–operative*_ – Inversion Effect_*post–operative*_). If the reduction in inversion effect of a patient was higher than one standard deviation of the inversion effect under the pre-operative condition of all patients, the patient was diagnosed with SCD.

### Statistical Analysis

After diagnosing SCD, the performance of the BM perception task was further analyzed by performing a repeated measures mixed-design ANOVA, taking time (pre-operative vs. post-operative) and orientation (upright *vs.* inverted) as the within-subject factor, and group (SCD vs. non-SCD) as the between-subject factor. The inversion effect of BM was calculated by comparing the performance between upright and inverted conditions (*d*′_*upright*_- *d*′_*inverted*_). The MMSE scores were analyzed by performing a paired *t*-test, taking time as the factor of interest (pre-operative vs. post-operative).

To examine whether the SCD was indeed independent from POCD, we analyzed the correlation between SCD and general cognition deficit (delayed neurocognitive recovery). Pearson correlation analyses were conducted between the MMSE score and the *d*′ of BM detection. The alpha level for all tests was 0.05.

## Results

### BM Perception Performance

According to the 1SD diagnostic criterion, 10 (four males) of 32 patients (31.25%) met the criterion of SCD, and 22 patients (14 males) had intact social ability. According to this criterion, patients were divided into two groups.

**FIGURE 2:**
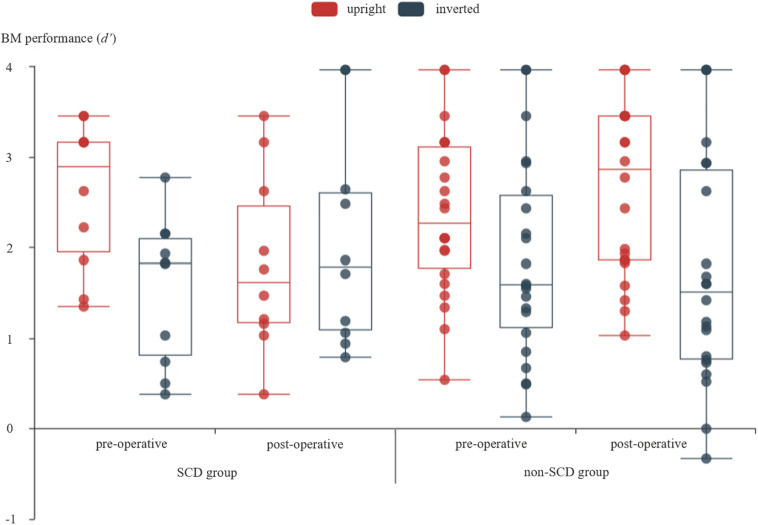
The detection of BM under different measure times and different orientations of BM stimuli for SCD and non-SCD group, respectively. For each box plot, the top line showed the maximum of the data, the box means the range from third quartile to first quartile, the line inside the box showed the median, the bottom line showed the minimum of the data, and there was no outlier according to the 1.5 inter-quartile range criterion. The scatter figure showed the data from every patient.

Figure 2 shows performance of BM perception task for every participants in every conditions. The mixed three-way ANOVA revealed a significant main effect of orientation [*F*(1,30) = 12.303, *p* = 0.001, $\eta_{\text{p}}^{2}$ = 0.291], suggesting that the BM perception under the upright condition was substantially better than the inverted condition across all the patients, and the inversion effect of BM (Sumi, 1984; Pavlova and Sokolov, 2000) was replicated. The main effect of time was not significant [*F*(1,30) = 0.029, *p* = 0.867, $\eta_{\text{p}}^{2}$ = 0.001], suggesting that the overall performance on BM perception did not evidently change after surgery. The main effect of the group was not significant [*F*(1,30) = 0.211, *p* = 0.649, $\eta_{\text{p}}^{2}$ = 0.007], implying that the performance of BM perception was comparable between groups. There was a significant interaction between time and orientation [*F*(1,30) = 8.592, *p* = 0.006, $\eta_{\text{p}}^{2}$ = 0.223]. The simple effect analysis revealed that the detection of BM was significantly larger for upright BM (*d*′ = 2.477) than for inverted BM (*d*′ = 1.694) before the surgery (*p* < 0.001) across all the patients. However, there was no significant difference between the upright (*d*′ = 2.237) and inverted (*d*′ = 1.896; *p* = 0.083) BM after the surgery across all the patients. In addition, the detection of upright BM was comparable between before and after surgery (*p* = 0.096), so was the detection of an inverted BM (*p* = 0.134). Neither the time × group interaction [*F*(1,30) = 0.783, *p* = 0.383, $\eta_{\text{p}}^{2}$ = 0.025], nor the orientation × group interaction [*F*(1,30) = 0.918, *p* = 0.346, $\eta_{\text{p}}^{2}$ = 0.030] reached significance.

Critically, a significant time × orientation × group interaction was revealed [*F*(1,30) = 32.090, *p* < 0.001, $\eta_{\text{p}}^{2}$ = 0.517]. This interaction was elaborated by performing a two-way repeated measure ANOVA for SCD and non-SCD, separately. For the SCD group, a significant main effect of orientation was revealed [*F*(1,9) = 6.888, *p* = 0.028, $\eta_{\text{p}}^{2}$ = 0.434]. The main effect of time was not significant [*F*(1,9) = 0.299, *p* = 0.598, $\eta_{\text{p}}^{2}$ = 0.032]. Critically, the interaction between time and orientation was significant [*F*(1,9) = 182.362, *p* < 0.001, $\eta_{\text{p}}^{2}$ = 0.953]. The simple effect analysis revealed a significant inversion effect before surgery by showing a higher *d*′ for upright BM (*d*′ = 2.586) than for inverted BM (*d*′ = 1.530; *p* < 0.001). However, the inversion effect was slightly reversed after surgery by showing an a slightly higher *d*′ for the inverted BM (*d*′ = 2.059) comparing to the upright BM (*d*′ = 1.820; *p* = 0.206). In addition, the detection of upright BM was significantly reduced in the post-operative condition relative to that in the pre-operative condition (*p = 0*.006), while the detection of inverted BM was even significantly enhanced (*p* = 0.047). For the non-SCD group, the ANOVA revealed a significant main effect of orientation [*F*(1,21) = 12.449, *p* = 0.002, $\eta_{\text{p}}^{2}$ = 0.372]. The main effect of time was not significant [*F*(1,21) = 0.482, *p* = 0.495, $\eta_{\text{p}}^{2}$ = 0.022]. The interaction between time and orientation was significant [*F*(1,21) = 4.378, *p* = 0.049, $\eta_{\text{p}}^{2}$ = 0.173]. A simple effect analysis revealed that the inversion effect was significant both in the pre-operative condition (*p* = 0.023) and post-operative condition (*p* = 0.001), suggesting that the global processing of BM took place, regardless of the surgery. In addition, there was no significant difference between the pre-operative and post-operative conditions in both the detection of upright BM (*p* = 0.089) and inverted BM (*p* = 0.389).

### Delayed Neurocognitive Recovery and Its Relation to SCD

A paired *t-*test revealed a significant difference between the pre-operative condition (*M* = 25.81, *SD* = 2.63) and post-operative condition (*M* = 24.63, *SD* = 3.32) on the MMSE score [*t*(31) = 2.552, *p* = 0.016, Cohen’s *d* = 0.45], indicating that the overall MMSE score significantly decreased after the surgery. According to the 1SD diagnostic criterion, 10 of 32 patients (31.25%) had delayed neurocognitive recovery, which was largely in line with previous studies which showed a prevalence range of 20–60% for elderly patients with cardiac surgery (e.g., Bekker and Weeks, 2003). Moreover, the independent *t*-test revealed that there was no group difference of MMSE decrease between the SCD group and non-SCD group [*t*(30) = 0.447, *p* = 0.658, Cohen’s *d* = 0.17].

**FIGURE 3:**
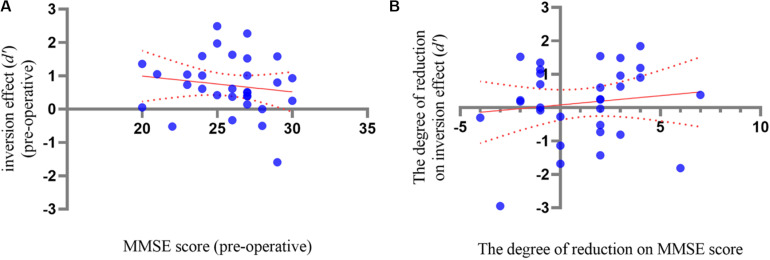
**(A)** The correlation analysis between inversion effect of BM and the MMSE score in pre-operative phase. **(B)** The correlation analysis between the inversion reduction and MMSE decrease between pre- and post-operative phases. The dotted lines showed 95% CI of simple linear regression.

For the correlation between delayed neurocognitive recovery and SCD, the Pearson correlation analysis revealed a non-significant correlation between pre-operative MMSE score and the inversion effect of BM in the pre-operative phase, *r* = −0.163, *p* = 0.373 (Figure 3A). A Pearson correlation analysis between MMSE score decrease and reduction of inversion effect was further conducted, and did not reach significance, *r* = 0.105, *p* = 0.567 (Figure 3B). These results suggested that there was no correlation between SCD and delayed neurocognitive recovery.

### Comparison Between SCD and Non-SCD on Demographics and Medical History

The demographics and medical history between SCD and non-SCD were compared by conducting independent *t*-tests and Fisher’s exact test. The results were illustrated in Table 1. None of difference between the SCD group and non-SCD group in the demographics or medical factors reached significance.

**TABLE 1:** Comparison of the demographics and medical history (mean ± SD) of patients.

	All patients (32)	SCD (10)	Non-SCD (22)	Comparison between SCD and non-SCD
Age (years)	66.8 ± 4.8	66.6 ± 3.1	66.9 ± 5.4	*t*(30) = 0.139, *p* = 0.890
Education level (years)	9.3 ± 3.4	8.7 ± 3.7	9.5 ± 3.2	*t*(30) = 0.640, *p* = 0.550
Gender (male/female)	18/14	4/6	14/8	*p* = 0.267
Diabetes	6.25%	20%	0%	*p* = 0.091
Hypertension	43.75%	40%	45.45%	*p* = 1.000
History of smoking	40.63%	20%	50%	*p* = 0.141
History of alcohol consumption	21.88%	10%	27.2%	*p* = 0.387
Surgery duration (min)	221.1 ± 61.7	193.0 ± 54.6	233.9 ± 60.5	*t*(30) = 1.769, *p* = 0.087
CPB duration* (min)	101.5 ± 44.4	93.1 ± 30.7	105.5 ± 49.7	*t*(29) = 0.853, *p* = 0.475
Sufentanil (μg)	164.8 ± 59.5	145.5 ± 49.4	178.5 ± 61.8	*t*(30) = 1.231, *p* = 0.228
Midazolam (mg)	8.8 ± 4.1	8.2 ± 3.9	8.8 ± 4.3	*t*(30) = 0.492, *p* = 0.626
Anesthesia time (min)	251.3 ± 70.0	228.9 ± 54.3	244.8 ± 60.6	*t*(30) = 1.212, *p* = 0.235
ICU duration (day)	4.0 ± 1.3	4.2 ± 1.6	3.7 ± 1.3	*t*(30) = 0.551, *p* = 0.585
Time to discharge (day)	10.2 ± 2.9	11.0 ± 3.0	10 ± 3.2	*t*(30) = 1.062, *p* = 0.297

*Since one patient skipped CPB progress, the analysis of CPB duration excluded this patient.

## Discussion

The present study examined whether social cognitive function could be impaired for elderly patients after anesthesia and surgery. All participants could detect BM from dynamic noise before surgery. Moreover, both the SCD and non-SCD group exhibited a clear inversion effect of BM before surgery. However, the inversion effect vanished or even slightly reversed for SCD after surgery, while the inversion effect was even larger for the non-SCD group. Consequently, we offered the first empirical evidence showing that SCD occurred after anesthesia and surgery. We demonstrated that 31.25% of patients exhibited impaired BM processing relative to that before anesthesia and surgery. Moreover, the correlation analysis revealed that the SCD was independent from delayed neurocognitive recovery, highlighting the necessity of paying attention to SCD, particularly considering that human beings are extensively social species.

The present study contributed to fully understanding the impact of surgery on the processing in human brain. Our finding was congruent with the implication of previous clinical research on social cognitive dysfunction (Christidi et al., 2018), offering the first clear-cut empirical evidence that BM perception is impaired in a group of patients after surgery. Moreover, the present study revealed that the impaired BM perception in SCD was rooted in the impairment of the global processing for BM, by showing that the inversion effect vanished after the surgery in SCD. In other words, the SCD patients could not distinguish upright BM from inverted BM. Furthermore, the data revealed that the SCD patients’ perception of upright BM dramatically impaired after the surgery, while their detection of inverted BM enhanced. Those finding implied that the SCD patients transformed the processing of BM from a global manner to a sophisticated local manner. Someone may argue that the observed SCD was due to a practice effect. We considered that this alternative did not hold (Krenk et al., 2012). Particularly, the BM test was firstly conducted 1 day before the surgery, and was performed the second time 7 days after the surgery. Therefore, there were 8 days between the two times of experiment. It is hard to imagine that the practice effect from a very simple perception task could linger 8 days. Moreover, supposing the practice effect could explain the raised performance of inverted stimuli after surgery (a difficult condition), it is difficult to explain why the practice effect would impair the performance of upright stimuli (an easy condition).

The present study was also the first to explore BM perception on patients after anesthesia and surgery. No study had tested the BM perception for patients after anesthesia and surgery. Compared to the traditional measurement of social cognition *via* questionnaire (e.g., Empathy Quotient, Baron-Cohen and Wheelwright, 2004), the present BM perception task was easy to perform, and user-friendly to patients (certain patients even considered the present task as a game). Future studies may consider using the present task to measure the status quo of their social cognition in a convenient manner.

Considering that delayed neurocognitive recovery and SCD occurred independently, the conclusions drawn from delayed neurocognitive recovery cannot be directly deduced to SCD. Therefore, a set of important issues should be investigated in future studies. For instance, as the first study that empirically explored SCD, the present study diagnosed the SCD following a criterion used in delayed neurocognitive recovery. As aforementioned, there were at least two ways in diagnosing delayed neurocognitive recovery (Evered et al., 2011). Additional studies are needed to determine the appropriate criterion for SCD diagnosis. In addition, it remains unclear that how long does the SCD last, and whether the type of surgery affects the life-span of SCD in studies on delayed neurocognitive recovery (see Evered et al., 2011 for an example). Moreover, future studies are needed to determine the potential affecting factors that influence SCD. This issue has been extensively explored in delayed neurocognitive recovery and POCD (Funder and Steinmetz, 2012).

Finally, three limitations of the current study should be noted. First, since the present study focused on cardiac surgery, it remained unknown whether the present finding could be extended to other surgery types, which requires to be addressed in future studies. It has been revealed that the type of surgery affected the type of cognition dysfunction in rats (Hovens et al., 2015). For instance, cardiac surgery reduced the performance in spatial learning and object recognition, while non-cardiac surgery did not have an effect. Second, the relatively small sample size may reduce the stability of our finding. Due to the limitation of accessibility of participants, we only collected 46 participants in total (32 valid) in almost 10 months. Therefore, although our study implied that SCD occurs after cardiac surgery, future study had to test a large group of patients to reach an exact ratio estimation of the SCD. Third, although MMSE is a broadly used test for diagnosing cognitive dysfunction (Burns et al., 1998; Folstein and Folstein, 2010), recently researchers suggested that using a set of tests was better than merely using MMSE (Evered et al., 2018). Since neither POCD nor delayed neuro-cognitive recovery was the main concern of this study, we chose MMSE, a simple but robust measure, for diagnosing cognitive dysfunction. However, future study may consider using the new test battery to further compare POCD and SCD.

## Conclusion

The present study was the first to indicate that elderly patients with anesthesia and surgery could have deficit in BM processing, particularly, had incidence of 31.25% in cardiac surgery, suggesting that SCD occurred after anesthesia and surgery. Meanwhile, SCD was independent from delayed neurocognitive recovery.

## Data Availability Statement

The datasets generated for this study are available on request to the corresponding author.

## Ethics Statement

The study was approved by the Research Ethics Board of Zhejiang University, and was performed in accordance with the approved guidelines. The clinical trial registration number is ChiCTR1800019874. The date of registration is 3rd December, 2018. Written informed consent was obtained from all participants.

## Author Contributions

DZ, JY, XM, HC, and ZG conceived and designed the study. DZ, JY, XM, HC, XL, and SZ performed the experiments. DZ, JY, XM, XL, and ZG wrote the manuscript. All authors analyzed the data, reviewed the manuscript, and approved the final version of the manuscript for submission.

## Conflict of Interest

The authors declare that the research was conducted in the absence of any commercial or financial relationships that could be construed as a potential conflict of interest.
